# The effectiveness of virtual reality-based technology on anatomy teaching: a meta-analysis of randomized controlled studies

**DOI:** 10.1186/s12909-020-1994-z

**Published:** 2020-04-25

**Authors:** Jingjie Zhao, Xinliang Xu, Hualin Jiang, Yi Ding

**Affiliations:** 1 School of Media, Xijing University, Xian, China; 2 Department of Traumatic Surgery, Jining No.1 Peoples Hospital, Jining, China; 3 Health Science Center, Xi’an Jiaotong University, Xian, China; 4 Department of Pharmacy, Xijing Hospital, Fourth Military Medical University, Xian, China

**Keywords:** Augmented and virtual reality, Improving classroom teaching, Teaching/learning strategies

## Abstract

**Background:**

Virtual reality (VR) is an innovation that permits the individual to discover and operate within three-dimensional (3D) environment to gain practical understanding. This research aimed to examine the general efficiency of VR for teaching medical anatomy.

**Methods:**

We executed a meta-analysis of randomized controlled studies of the performance of VR anatomy education. We browsed five databases from the year 1990 to 2019. Ultimately, 15 randomized controlled trials with a teaching outcome measure analysis were included. Two authors separately chose studies, extracted information, and examined the risk of bias. The primary outcomes were examination scores of the students. Secondary outcomes were the degrees of satisfaction of the students. Random-effects models were used for the pooled evaluations of scores and satisfaction degrees. Standardized mean difference (SMD) was applied to assess the systematic results. The heterogeneity was determined by *I*^*2*^ statistics, and then was investigated by meta-regression and subgroup analyses.

**Results:**

In this review, we screened and included fifteen randomized controlled researches (816 students). The pooled analysis of primary outcomes showed that VR improves test scores moderately compared with other approaches (standardized mean difference [SMD] = 0.53; 95% Confidence Interval [CI] 0.09–0.97, *p* < 0.05; *I*^*2*^ = 87.8%). The high homogeneity indicated that the studies were different from each other. Therefore, we carried out meta-regression as well as subgroup analyses using seven variables (year, country, learners, course, intervention, comparator, and duration). We found that VR improves post-intervention test score of anatomy compared with other types of teaching methods.

**Conclusions:**

The finding confirms that VR may act as an efficient way to improve the learners’ level of anatomy knowledge. Future research should assess other factors like degree of satisfaction, cost-effectiveness, and adverse reactions when evaluating the teaching effectiveness of VR in anatomy.

## Background

Anatomy is a visual science which is thought an important foundation for medical learning [1]. When studying anatomy, the learners identify structures and their spatial relationships. Nonetheless, medical students often experience trouble acquiring adequate understanding of three dimensional (3D) anatomy from graphic images, such as those in textbooks and PowerPoint [2, 3]. So, it has become vital to create modern strategies concentrated on efficient as well as high-quality anatomy education and learning.

With new learning tools developing, the health and medical education system has started incorporating more interactive media and online materials. The utilization of computer-based 3D models in anatomy education has become a favorite over the last years [4]. Notably, VR is a technology that allows exploring and manipulating computer-generated real or artificial 3D multimedia environments in real-time. It allows for a first-person active learning experience through different levels of immersion. The rise of virtual reality technology could be traced back to the 1960’s in the entertainment industry. VR promises to provide more immersive, engaging experiences, with applications in many domains, including shopping, entertainment, training, and education [5]. Developers have created compelling experiences allowing people to travel through the cells of the body, to explore the Solar System, and to encounter recreations of ancient battles in history. Particularly, virtual reality technologies frequently were used for flight simulator training and exercises [6].

Recently, increasing interest has been paid to VR in the medical educational world, particularly for anatomy teaching and resident surgical training [7, 8]. VR provides students a simulation scene to conceptualize intricate 3D anatomic connections quickly. Some studies have compared VR to the other teaching methods for anatomy such as dissection, lectures, 2D images, and blended instruction. For example, in 2019 Maresky et al. tested the effectiveness of a VR simulation of the heart in medical teaching [9]. They found that students (*n* = 28) under the VR simulation performed significantly better than the control group (*n* = 14) in the final test. In 2015, a meta-analysis was conducted to evaluate teaching effect of using 3D visualization approaches in educational anatomy [10]. The results showed that 3D visualization methods are better teaching tools than 2D methods in the acquisition of factual anatomy knowledge and spatial anatomy knowledge. However, there is no high level of evidence on how efficient these different VR approaches are when contrasted to various other techniques in randomized controlled studies.

Accordingly, the purpose of this meta-analysis was to explored the educational effectiveness of VR applied to anatomy education in comparison with conventional or 2D digital methods in class.

Three research questions guided this study:

1. Are the test scores improved using VR education as compared to the other teaching methods?
2. Are the satisfaction levels higher in VR education as compared to the other teaching methods?
3. Do year of publication, country of study, subject of learning, intervention, comparator, and duration play a moderating role in the distinction?

## Methods

### Search strategy

This study adhered to the PRISMA criteria [11]. Search terms for OVID MEDLINE was firstly performed and after that adjusted for the others: Embase, Cochrane Central Register of Controlled Trials, Web of Science Core Collection, and clinical trial registries. Terms as well as subheadings such as key terms (anatomy) AND (virtual reality OR virtual learning environment OR mixed reality OR virtual classrooms OR augmented reality OR visualization technologies) AND (educat* OR simulat* OR training). Databases were searched from January 1990 to August 2019.

The search results from various databases were incorporated with Endnote software (EndNote X7, Clarivate Analytics, Philadelphia), and duplications of included studies were eliminated. Two authors (Y.D. and J.J.Z) separately screened the search results as well as examined full-text research studies for inclusion. Any kind of disputes, for unclear or missing information were settled via conversation between the authors.

### Inclusion and exclusion criteria

We included randomized controlled studies on comparing and studying VR intervention with control methods in anatomy teaching. In this review, VR methods including types of interactive 3D models, virtual patient or and surgical simulation could be performed as the single intervention or blended with others [12]. VR as an intervention for education can be displayed with a variety of tools, including computer or mobile device screens, and VR rooms of head-mounted displays. Studies were excluded with the following reasons: not randomized controlled study; not in the field of anatomy education, absence of an intervention; absence of test scores; insufficient data for effect size calculation. Exclusion was conducted by Y.D. and J.J.Z, and inconformity was discussed and resolved. The Kappa score was used to calculate the inter-investigator agreement during the inclusion process for publication-evaluated databases.

### Data extraction

We extracted data from validity studies according to the Cochrane Handbook for Systematic Reviews [11]. In this review, the main concerned information covered year and region of the publication, details of learners, interventions, and duration of the study. Both of authors (J.J.Z and Y.D.) assessed the risk of bias for randomized controlled trials by the Cochrane risk of bias tool [13].

### Data synthesis and heterogeneity assessment

All analyses were conducted by Stata 15 (StataCorp, College Station, TX, USA). Comparators included traditional education, other forms of digital education, and other types of VR. For continuous data of test scores and satisfaction levels, we summarized the standardized mean differences (SMDs) and associated 95% confidence interval (CI) across studies. We were unable to identify a clinically meaningful interpretation of SMDs for different kinds of VR education interventions. Therefore, the effect size was determined by the value of SMDs based on the Cohen rules: < 0.2 (none), 0.2 to 0.5 (small), 0.5 to 0.8 (moderate), and > 0.80 (large) [14]. We applied *I*^*2*^ statistic to determine heterogeneity. *I*^*2*^ < 25% (low), 25 to 75% (medium), and > 75% (high) indicate different levels of heterogeneity [15]. The fixed effect model was used to pool data if there was no heterogeneity (*I*^*2*^ > 50%); otherwise, the random effects model was used (*I*^*2*^ < 50%).

Subgroup analysis was conducted when feasible. Seven attributes of each random were coded as possible moderators: year, region, learners, course, intervention, comparator, and duration. Sensitivity analyses was conducted to determine if the individual study significantly altered the results of meta-analyses [16]. Publication bias was determined by a funnel plot [17] and Begg’s test [18]. The *p* value < 0.05 was defined as significant.

## Results

### Search results

**Fig. 1:**
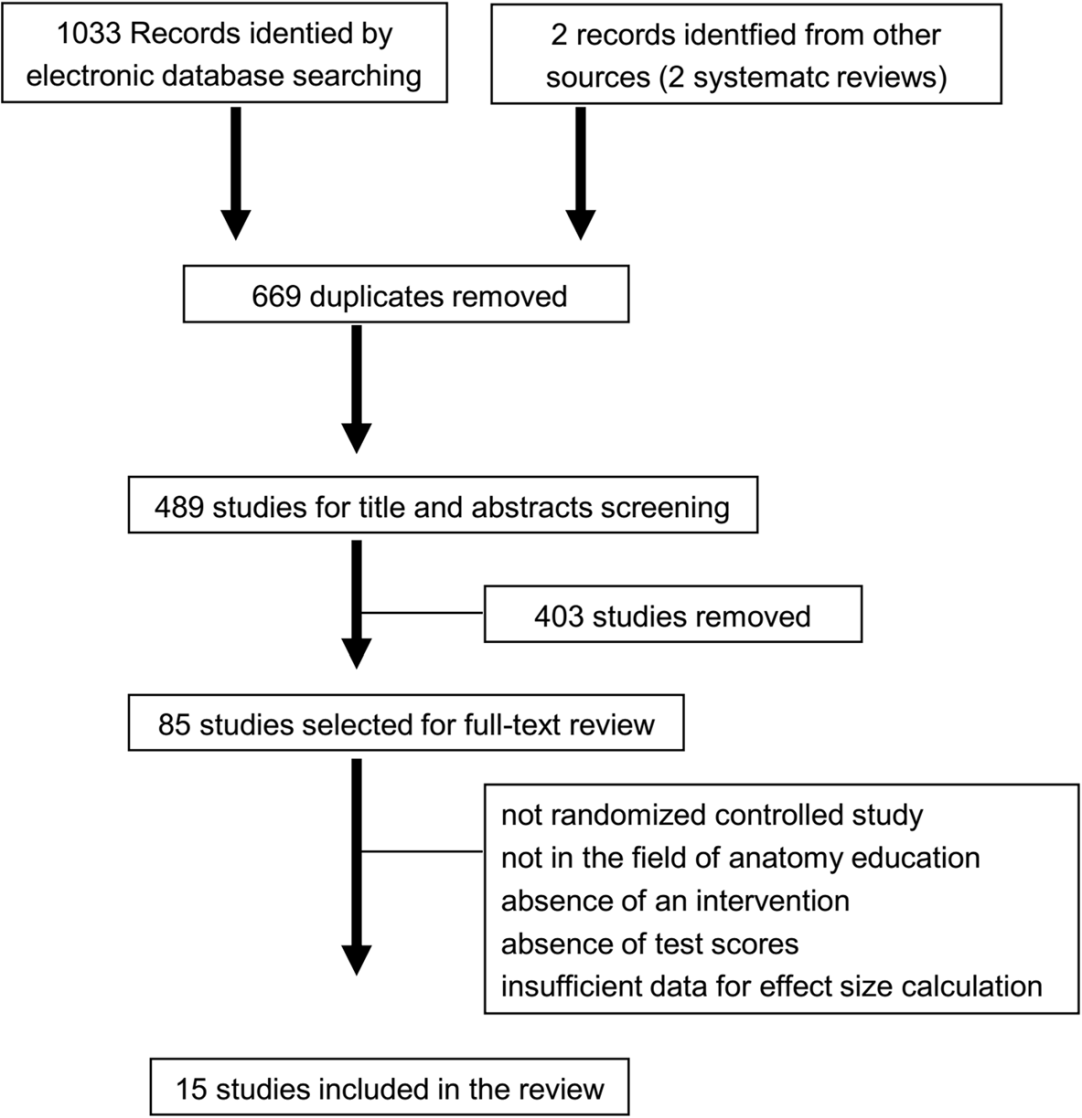
Flowchart of the search strategy

Overall, 15 studies met the inclusion requirements (Fig. 1 and Table 1). There were 15 randomized controlled studies with an overall of 816 learners: 745 were medical students and 71 were residents. There were seven studies performed in USA, two studies in UK, two studies in Canada, and one each in Brazil, Australia and Japan. A series of VR educational methods were evaluated, including interactive 3D models, VR or and VR surgical stimulations. Interventions in the control group ranged from traditional learning (lecture, dissection and/or textbooks) to other digital education interventions. The duration of the intervention varied between 10 min to 2 weeks. For all research studies, primary results were determined by evaluation or survey studies at the end. And five out of 15 studies assessing satisfaction levels as the secondary outcome [23, 24, 30, 31, 33]. Table 1 shows the study characteristics of involved studies.

**Table 1:** Characteristics of included studies

First author	Participants/Country	N (VR/control)	Course	Intervention	Comparator	Duration
Anthony, 2011 [19]	medical students/UK	12/14	anatomy of the forearm	VR	dissection and textbooks	50 min
Battulga, 2012 [20]	medical students/Japan	50/50	shoulder	3D interactive models	2D images	60 min
de Faria, 2016 [21]	medical students/Brazil	28/28	neuroanatomy	3D interactive models	2D images	60 min
Ellington, 2018 [22]	residents/UK	16/15	female pelvic anatomy	VR	power point	2 weeks
Hampton, 2010 [23]	medical students 3, 4 year /USA	21/22	female pelvic anatomy	3D interactive models	dissection and textbooks	60 min
Keedy, 2011 [24]	medical students 1, 4 year/USA	23/23	anatomy of the liver	3D interactive models	2D images	1 day
Khot, 2013 [25]	medical students/Canada	20/20	pelvic anatomy	VR	power point	10 min
Kockro, 2015 [26]	medical students/Germany	89/80	spatial neuroanatomy	3D interactive models	power point	20 min
Moro, 2017 [27]	medical students/Australia	20/22	skull anatomy	VR	3D models	10 min
Nicholson, 2004 [28]	medical students 1 year /USA	29/28	ear anatomy	3D interactive models	text books	2 day
Seixas, 2010 [29]	surgical trainees/USA	5/5	human anatomy	VR	2D images	1 day
Solyar, 2008 [30]	medical student/USA	7/8	paranasal sinuses	VR	textbooks	60 min
Stepan, 2017 [31]	medical students 1,2 year /USA	33/33	neuroanatomy	VR	text books	1 day
Tan, 2012 [32]	residents/ Canada	21/19	laryngeal anatomy	3D interactive models	text books	45 min
Zachary, 2015 [33]	medical students/USA	41/32	neuroanatomy	3D interactive models	2D images and 3D models	65 min

### Inter-investigator agreement

The inter-investigator agreement (Kappa) was calculated by evaluating the selected titles and abstracts, and then obtaining a value for selected articles (kappa = 0.92) presenting a high level of agreement between the reviewers under the Kappa criteria [14].

### Risk of bias assessment

**Fig. 2:**
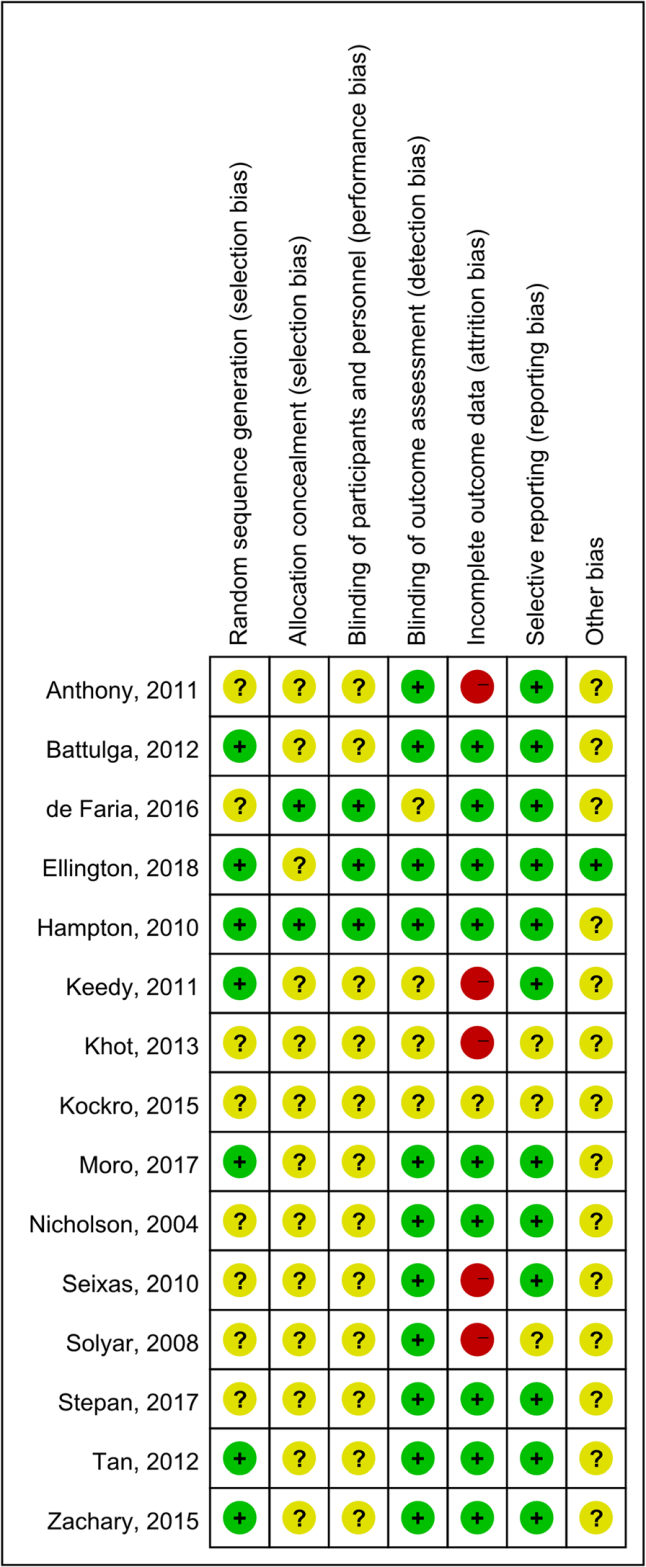
Risk of bias assessment of included studies

The risk of bias in majority of studies involved was unclear or high risk as shown in the bias summary (Fig. 2). Most studies did not have information about allocation concealment and baseline of learners’ characteristics. Due to the nature of the intervention, it is not practical for blinding of students and teachers during the study. For risk of completeness of data, and selective reporting, most studies were determined low. It was assessed whether the research study was devoid of selective outcome reporting, which checked whether outcomes mentioned adequately in manuscripts. Five studies were judged to be of high risk on completeness of data because of incomplete or accurate data on outcome standard deviation [19, 24, 25, 29].

### Data analysis

**Fig. 3:**
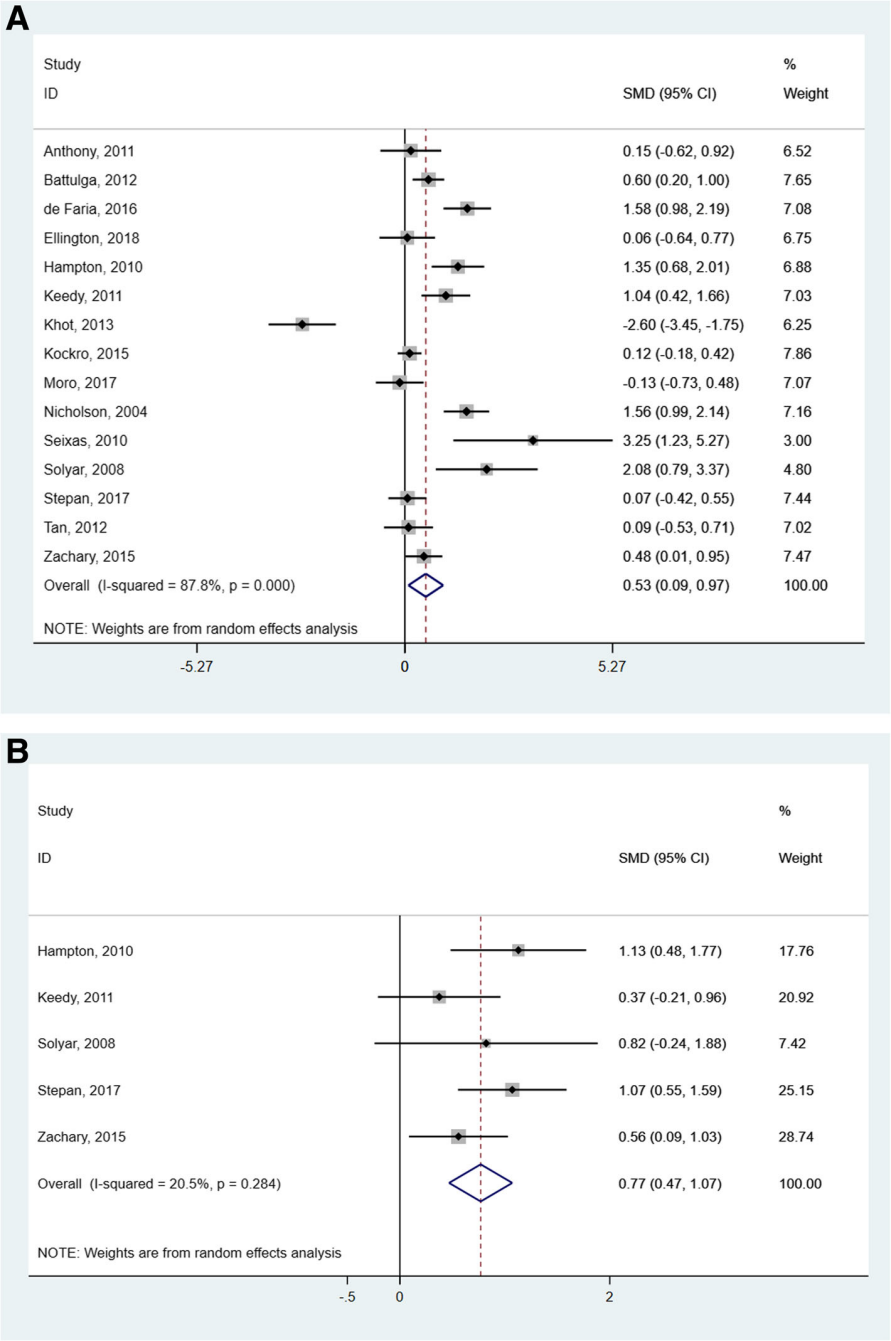
Forest plots for examination scores (**a**) and satisfaction outcomes (**b**)

The meta-analysis plots of primary and secondary outcomes are shown in Fig. 3a and b. The effectiveness of intervention on examination scores was reported in all studies. The studies assessed test scores as a primary outcome with multiple-choice questionnaires. We found that VR significantly increased learners’ examination scores compared with traditional learning in the random-effects model (SMD = 0.53; 95% CI 0.09–0.97, *p* < 0.05; *I*^*2*^ = 87.8%) (Fig. 3a). Nine of the studies (60%) showed that VR significantly increased students’ examination scores when compared with traditional learning (lecture, dissection and/or textbooks) to other digital 2D methods; and five (15%) failed to reveal statistically significant effects between the VR and the control groups. Outcomes showed that the studies were heterogeneous (*p* < 0.001) and the true effects were not consistent among studies.

A total of five studies assessed satisfaction levels as a secondary outcome [23, 24, 30, 31, 33]. The pooled results based on the fixed effects model showed that most students have a greater interest in learning via VR methods, rather than conventional or 2D teaching methods (SMD = 0.77; 95% CI 0.47–1.07, *p* < 0.05; *I*^*2*^ = 20.5%). However, only one study mentioned the adverse effects that some participants using VR displayed, including headaches, dizziness, or blurred vision [27].

### Publication bias

**Fig. 4:**
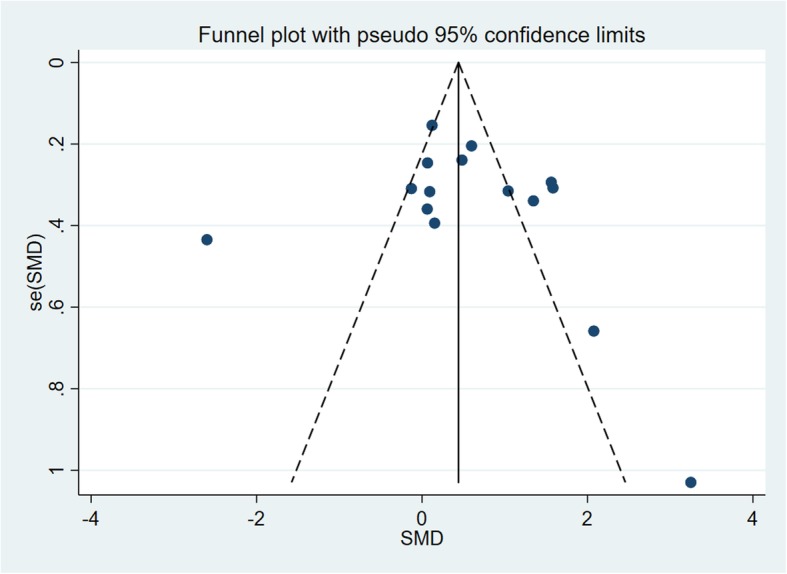
Funnel plot analysis for examination scores

For the primary analyses, funnel-plots were made to check for risk of publication bias (Fig. 4). The shape of the funnel plot was found to be symmetrical. Meanwhile, the result of Begg’s test show a non-significant asymmetry (*p* = 0.54) [34]. Thus, there was no significant publication bias indicated in this review.

### Subgroup analyses

A random-effects model was used for the subgroup analysis due to each subgroup being heterogeneous according to the results of tests (Table 2) [35]. As indicated in Table 2, the categorical variables were as follows: region (USA or others), learners (medical students or residents), course (skeletal anatomy or neuroanatomy or others), intervention (3D interactive models or VR simulations), comparator (traditional methods or other digital methods) and duration (< 1 day or ≥ 1 day). Other potential moderators could not be analyzed because they were reported inadequately to do a subgroup analysis. The differences in the subgroups for Q statistics are non-significant (*I*^*2*^ > 75%).

**Table 2:** Summary statistics for moderators related to examination scores

Subgroup	n	SMD	95% CI	***p*** value	I^**2**^
**region**					
USA	7	1.14	0.56, 1.72	0.00	79.8%
others	8	0.03	−0.57, 0.63	0.92	89.6%
**learners**					
medical students	12	0.51	0.02, 1.01	0.04	89.6%
residents	3	0.67	−0.45, 1.79	0.24	77.8%
**course**					
skeletal anatomy	6	−0.07	−0.95, 0.81	0.88	91.4%
neuroanatomy	4	0.52	−0.04, 1.10	0.07	84.9%
others	5	1.34	0.52, 2.14	0.00	87.8%
**intervention**					
3D interactive models	8	0.64	0.47, 0.81	0.00	82.5%
VR	7	−0.09	−0.37, 0.18	0.50	89.2%
**comparator**					
traditional methords	5	0.81	0.15, 1.47	0.02	82.6%
other digital methods	10	0.35	−0.25, 0.95	0.25	90.2%
**duration**					
< 1 day	10	0.35	0.18, 0.52	0.00	89.4%
≥1 day	5	0.71	0.42, 1.10	0.00	84.4%

Interestingly, the moderator analysis revealed significant benefits of VR in the subgroup of medical students (SMD = 0.51; 95% CI 0.02–1.01, *p* = 0.04), whereas VR have no significant influence on residents (SMD = 0.67; 95% CI -0.45–1.01, *p* = 0.24). Also, moderator analysis of control type showed that test scores of the VR group was not significantly better than using other 2D digital methods (SMD = 0.35; 95% CI -0.25–0.95, *p* = 0.25), while there was a significant improvement when compared with the traditional intervention group (SMD = 0.81; 95% CI 0.15–1.47, *p* = 0.02). For the duration analysis, VR interventions for at least 1 day had moderately-to-large effects on scores (SMD = 0.71; 95% CI 0.42–1.10, *p* < 0.001), whereas those which were <  1 day had only a small effect (SMD = 0.35; 95% CI 0.18–0.52, *p* < 0.001).

### Meta-regression analyses

To determine whether there were any moderation effects on primary outcomes, meta-regression analyses were conducted. We regressed effect sizes on 7 potential moderators: year, country, learners, course, intervention, comparator, and duration. As shown in Table 3, none of the moderators were significant at a level of *p* < 0.05.

**Table 3:** Meta-regression analysis for exploration of the sources of heterogeneity factors

Factors	Coefficient	Standard error	95% CI	***p*** value
year	−0.12	0.20	−3.06, 0.67	0.21
country	−1.19	0.95	−2.99, 0.54	0.17
learners	1.08	1.24	−1.35, 3.52	0.38
course	−0.26	0.89	−2.01, 1.49	0.77
intervention	−0.33	0.79	−0.53, 0.27	0.67
comparator	0.29	0.86	−1.40, 1.99	0.73
duration	0.09	0.95	−1.77, 1.97	0.91

### Sensitivity analyses

**Fig. 5:**
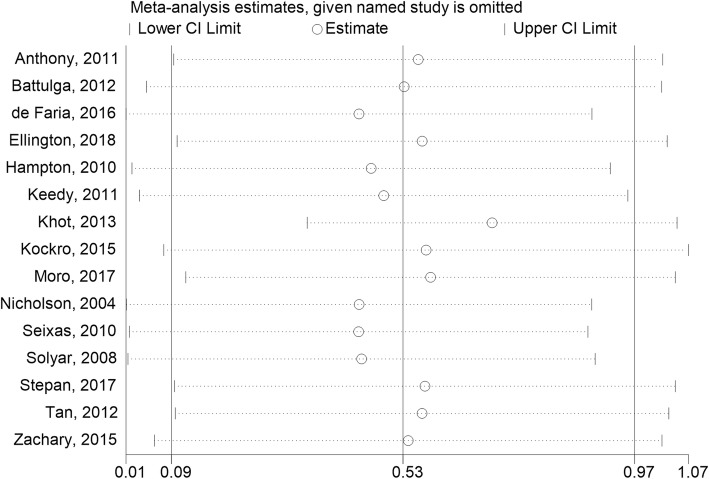
Sensitivity analysis assessing the influence of each study on the pooled analysis

Due to the significant heterogeneity (> 75%), a sensitivity analysis was used to verify the reliability of the result. When any research was removed from the model, the significant results of the VR effect on examination scores were unchanged in the models (SMD = 0.53, 95% CI: 0.01–1.07) (Fig. 5). Thus, the results indicated that the findings for examination scores were robust.

## Discussion

This meta-analysis of randomized controlled studies was conducted to examine the effectiveness of VR-based technology in anatomy teaching. We found that VR interventions have a moderate enhancement (SMD = 0.53) in test scores of learners in comparation with conventional or other 2D digital methods (*p* < 0.01). As has been previously found, more interactive interventions could moderately improve medical learners’ academic scores in anatomy [36]. Among 15 studies, only five studies assessed satisfaction scores as a secondary outcome with a result that most of students more interested in using VR to learn anatomy. Naturally, the fact that no included randomized controlled studies were found in databases before 2004 suggested that VR was an emerging academic method [37], attracting increasing interest from the world of education. In general, the risk of bias for most studies was unclear for a lack of description or data. Potentially high risk of incomplete reporting bias was identified in some studies. However, results of sensitivity and subgroup analyses were nonsignificant for variables (year, country, learners, course, intervention, comparator, and duration) on the outcome variables. Since the different types of learners and interventions in researches in this review, inconsistent methodological method makes it difficult to draw accurate conclusions.

In the subgroup analysis for levels of learners, the source of high heterogeneity could be diverse phases of participants’ medical education among included studies. Learners are first-year medical students in two studies [28, 31], while learners in another two studies are forth-year medical students [23, 24]. Of course, the longer learners acquired more knowledge of anatomy, which leads to comparing results complex or paradoxical. As Hattie et al. had concluded in 2015, the different degrees of expertise of learners are remarkable in education [38]. Therefore, medical students could be more easily motivated and effective in front of the fictitious scenarios of VR because they have fewer clinical experiences compared to residents. In addition, various organs or body parts learned present different levels of complexity, leading to the heterogeneity in results. For example, learning the anatomy of the brain was demonstrated harder than learning skeletal parts [31, 33]. In terms of duration, the results of this review showed that a course for 1 day or longer had a larger effect size than a course for several hours (0.71 vs 0.35). Thus, the learning duration has influenced the educational efficiency of VR methods, which should be considered and adjusted in practice.

Types of comparator is another source of variation. Only five of 15 studies were found where this technology was compared to traditional methods such as lectures, dissection or textbooks. However, it would be more meaningful to conduct evaluations of studies that compare the different features of digital-based methods rather than those which compare digital-based to traditional methods [19]. Dissection is regarded as the standard teaching method for anatomy. In this review, only two of 15 studies compared VR with dissection for anatomy teaching [19, 23]. In fact, VR could be used as an adjunct to dissection in class with fewer lab hours or resources. For example, in 2006 SN Biasutto et al. demonstrated that the best possibility in teaching anatomy is the correct association of cadaver dissections and computerized resources based on their studies [39].

For satisfaction scores, the pooled results of the comparison of VR versus others was significantly in favor of VR, which could be due in part to the novelty of the method. Most of the participants in the studies reported that the VR methods were easier and more enjoyable to use. In 2011, researchers had revealed that there was a significant positive correlation between motivation and academic record of students [40]. However, due to the complicated anatomical configuration, in one study, one third of participants found the VR methods disorienting and frustrating [27]. Using virtual reality could result in cybersickness, such as nausea, disorientation and headache [41]. Thus, more studies should focus on the adverse effects such as blurred-vision and disorientation caused by VR.

As a fast-moving technology, the cost of VR will be a critical aspect when considering to apply it into education especially for low-income settings. In this review, only one study is from a lower income setting [21], which reduces the applicability of innovative educational methods to developing regions. Unfortunately, no randomized controlled studies reported on cost-effectiveness of VR compared with other teaching methods.

### Strengths and limitations

VR is currently a new visualization technique, so there was no high-quality evidence on the effectiveness of VR-based technology. It is hard to offer an overall conclusion of the efficacy of these strategies. The strengths of this meta-analysis included detailed search on randomized controlled studies, and the data was drawn out by two of authors independently. Because of the variability in studies, we also assessed the risk of bias, sensitivity analyses and meta-regression analyses on outcomes from articles. Results of sensitivity and subgroup analyses were nonsignificant, indicating that the findings were robust.

This review also has several limitations. First, the included researches mainly reported post-intervention information, so we did not compute pre-to post-intervention modification. The validity of the different assessments used in the included studies might constitute a bias. Gender information was not easy to obtain in the current meta-analysis, but it is an important factor influencing teaching effect [42]. In future studies, information on gender ratio for treatment and control group may be collected for analysis. Another limitation was that, none of the studies assessed the cost of setup and maintenance of the VR-based intervention. Further research should evaluate the effectiveness of VR in a variety of settings and evaluate outcomes such as attitude, adverse effects, and cost-effectiveness.

## Conclusions

As an emerging and new technology, VR has the potential in transforming medical teaching. In this meta-analysis, results showed that when compared with traditional or 2D digital methods, VR can potentially improve teaching effectiveness of anatomy. However, our results are not certain for lack of standardized measures and high heterogeneity among studies, and the appropriate mode of integrating VR into class needs to be further explored. To enhance the teaching quality, VR as an implement could be considered on the medical teaching situations by universities and hospitals.

## Data Availability

The datasets used and/or analysed during the current study are available from the corresponding author on reasonable request.

## Abbreviations

CI: Confidence Interval
VR: Virtual reality
SMD: Standardized mean difference
3D: three-dimensional
